# Direct oral anticoagulants versus low-molecular-weight heparins for the treatment of acute venous thromboembolism in patients with gastrointestinal cancer: a systematic review and meta-analysis

**DOI:** 10.1186/s12959-022-00399-7

**Published:** 2022-07-28

**Authors:** Tarinee Rungjirajittranon, Weerapat Owattanapanich, Yingyong Chinthammitr, Theera Ruchutrakool, Bundarika Suwanawiboon

**Affiliations:** Division of Hematology, Department of Medicine, Faculty of Medicine Siriraj Hospital, Mahidol University, Bangkok, Thailand

**Keywords:** Acute treatment, Direct oral anticoagulants, Gastrointestinal cancer, Low-molecular-weight heparin, Patients, Venous thromboembolism

## Abstract

**Background:**

The association between gastrointestinal (GI) cancer and a high incidence of venous thromboembolism (VTE) is well known. Previous randomized controlled studies demonstrated that direct oral anticoagulants (DOACs) effectively treat cancer-associated thrombosis (CAT). However, some DOACs appeared to increase the risk of bleeding, particularly in patients with GI malignancies. Therefore, the current systematic review and meta-analysis were conducted to evaluate the safety and efficacy of DOACs in GI cancer-associated thrombosis.

**Methods:**

Two investigators individually reviewed all studies that compared DOACs and low-molecular-weight heparins (LMWHs) in GI cancer-associated thrombosis and were published in MEDLINE and EMBASE before February 2022. The effect estimates and 95% confidence intervals (CIs) from each eligible study were combined using the Mantel–Haenszel method.

**Results:**

A total of 2226 patients were included in the meta-analysis. The rates of major bleeding in the DOAC and LMWH groups were not significantly different (relative risk [RR]: 1.31; 95% CI: 0.84–2.04; *P* = 0.23; I^2^ = 41%). However, the rate of clinically relevant nonmajor bleeding (CRNMB) was significantly higher in the DOAC group (RR: 1.76; 95% CI: 1.24–2.52; *P* = 0.002; I^2^ = 8%). The risks of recurrent VTE in the groups did not significantly differ (RR: 0.72; 95% CI: 0.49–1.04; *P* = 0.08; I^2^ = 0%).

**Conclusions:**

The current data suggest that treatment of GI cancer-associated thrombosis with DOACs significantly increases the risk of CRNMB. However, the risk of major bleeding was not significantly different. The efficacy of DOACs for preventing recurrent VTE in GI cancer was comparable to that of LMWHs.

**Trial registration:**

INPLASY202180113.

**Supplementary Information:**

The online version contains supplementary material available at 10.1186/s12959-022-00399-7.

## Background

The relationship between cancer and thrombosis is well recognized. A recent population-based study showed that the cumulative incidence of venous thromboembolism (VTE) after cancer diagnosis was 11.1-fold higher than that in noncancer patients [1]. Moreover, VTE is among the leading causes of death in cancer patients [2]. The absolute rate of VTE in all cancers from a large United Kingdom database was 13.9 per 1000 person-years [3, 4]. A study in the East Asian population revealed an incidence of cancer-associated VTE of 9.9 per 1000 person-years in hepatocellular and pancreatic cancers [5].

In addition to ethnicity and cancer stage, the type of cancer also influences the risk of thrombosis. Gastrointestinal (GI) cancer (cancers of the pancreas, stomach, liver, colon, and rectum) is among the top 4 most prevalent cancers worldwide [6, 7]. A higher incidence of VTE was found in patients with GI cancer than in those without GI cancer [8, 9]. Singh R et al.reported that 60 of 220 (27.3%) patients with GI cancer experienced 83 thromboembolic events, including 38.6% deep vein thrombosis and 20.5% pulmonary embolism [9]. Interestingly, some of those patients experienced more than 1 thrombotic event, and some thromboses were incidentally found [9].

The treatment of cancer-associated thrombosis has vastly improved in recent years. Direct oral anticoagulants (DOACs) have become a standard treatment for VTE in patients with cancer. Their use is based on evidence from randomized controlled studies that compared the efficacy and safety of DOACs and low-molecular-weight heparins (LMWHs) [10–13]. Even though the benefit of DOACs in preventing recurrent thrombosis has been demonstrated in patients with cancer, the risk of bleeding is a drawback, especially in patients with GI malignancies. The Hokusai VTE Cancer trial found that major bleeding events among patients with GI cancer treated with edoxaban were significantly more frequent than for the dalteparin arm (13.2% *vs* 2.4%; *P* = 0.0169) [10]. In the SELECT-D study, patients with esophageal or gastroesophageal cancer receiving rivaroxaban tended to experience more major bleeding than those treated with dalteparin (36% *vs* 5%). Consequently, the recruitment of patients with this tumor type was stopped in the ongoing trial [11]. In contrast, the incidence of bleeding events, particularly in patients with GI malignancies, did not significantly differ between the apixaban and dalteparin arms in the ADAM VTE and Caravaggio trials [12, 13].

The present systematic review and meta-analysis aimed to improve our understanding of the efficacy and safety of DOACs in treating acute VTE in patients with GI cancer compared with LMWHs. To this end, a comprehensive identification was made of all available studies, and their data were summarized and analyzed.

## Methods

### Data sources and searches

All relevant studies that compared DOACs and LMWHs in GI cancer-associated thrombosis and were published before February 2022 were identified in 2 databases (MEDLINE and EMBASE). The search terms were “DOACs,” “anticoagulants,” and “GI cancer” (Additional file 1: Supplementary Data 1). Two investigators (TR and WO) separately examined the included articles. The Preferred Reporting Items for Systematic Reviews and Meta-Analyses Statement guided the meta-analysis (Additional file 2: Supplementary Data 2) [14]. The study protocol was registered with the International Platform of Registered Systematic Review and Meta-Analysis Protocols (registration number INPLASY202180113).

### Selection criteria and data extraction

The inclusion criteria for this meta-analysis were as follows: (1) the type of study must have been a randomized controlled trial (RCT) or a cohort study (either retrospective or prospective); (2) the study must have compared the efficacy of at least 1 DOAC and at least 1 LWMH in GI cancer-associated venous thromboembolism; (3) the study must have included the primary outcome; and (4) the study must have defined “major bleeding” according to the criteria of the International Society on Thrombosis and Haemostasis (ISTH) [15].

The same 2 investigators (TR and WO) independently selected relevant articles and extracted data. If there was any disagreement or question regarding the eligibility of an article, a third investigator (BS) made the final decision. The 2 investigators (TR and WO) examined the baseline characteristics data and the outcomes of all included studies. The extracted data were cross-checked to avoid inaccuracies.

### Outcome definitions

The primary outcome was either recurrent VTE or major bleeding after anticoagulant therapy, as defined by the ISTH criteria [15]. “Major bleeding” encompassed fatal bleeding, symptomatic bleeding in a critical area or organ, and bleeding causing a decrease in hemoglobin level of ≥ 2 g/dL or leading to the transfusion of ≥ 2 units of whole blood or red cells [15].

The secondary outcome was clinically relevant nonmajor bleeding (CRNMB). The studies in this meta-analysis used a variety of definitions of CRNMB. They are detailed in Additional file 3: Supplementary Data 3.

### Quality assessment

The “Cochrane Risk-of-Bias Tool for Randomized Trials” (ROB-2) [16] and the “Risk of Bias in Non-Randomized Studies of Interventions” (ROBINS-I) [17] were used to evaluate the quality of the included studies.

### Statistical analysis

Review Manager 5.3 software from the Cochrane Collaboration (London, UK) was used to analyze the data. Two investigators (TR and WO) extracted data from the selected studies using a standardized data extraction form. The effect was estimated and combined with 95% confidence intervals (CIs) using the Mantel–Haenszel method [18]. Cochran’s Q test was calculated, and the statistical heterogeneity among the studies was estimated using the I^2^ statistic. The 4 levels of heterogeneity were based on the value of I^2^ as follows: (1) insignificant heterogeneity (values of 0%–25%); (2) low heterogeneity (values of 26%–50%); (3) moderate heterogeneity (values of 51%–75%); and (4) high heterogeneity (values of 76%–100%) [19]. The random-effects model was applied based on the assumption that there was heterogeneity in the studies due to differing patient characteristics, DOACs, and types of GI cancers [19]. A probability (*P*) value less than 0.05 was considered statistically significant.

### Subgroup analyses

Subgroup analyses were based on the type of study to avoid heterogeneity and bias. Moreover, to determine the differences in bleeding risks and VTE recurrence related to each type of GI cancer and DOAC, we analyzed subgroups of patients according to GI cancer (luminal or nonluminal) and DOAC subtype.

## Results

### Study identification and selection

An electronic search of the MEDLINE and EMBASE databases revealed 1279 potentially relevant articles. After excluding 170 duplicate articles, 2 investigators reviewed the titles and abstracts of the remaining 1109 articles. Of those, 1069 articles were excluded if they met at least 1 of the following 3 criteria:

- 1. The articles were reviews, meta-analyses, commentaries, or editorials.
- 2. The reports were irrelevant to the comparison between DOACs and LMWHs.
- 3. The reports described a study population different from that evaluated in our study.

**Fig. 1:**
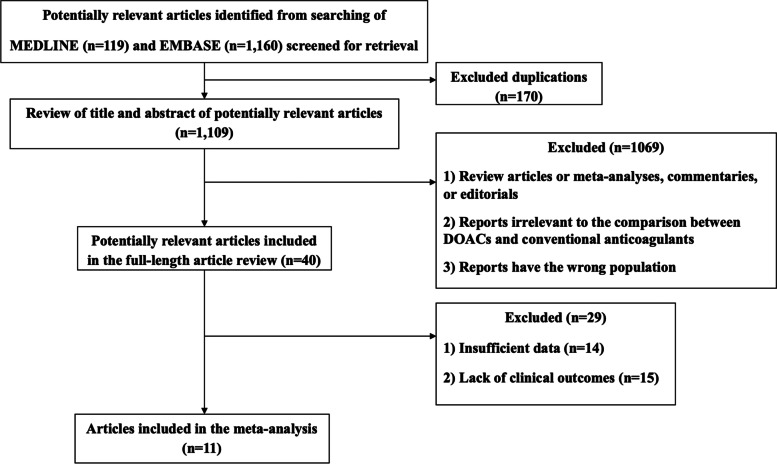
Flowchart of the literature review and article selection process

A total of 40 full-length articles were identified. Of those, 29 articles were excluded due to insufficient data or a lack of clinical outcomes. The remaining 11 articles (6 RCTs and 5 retrospective studies) collectively enrolled 2226 patients. Six articles evaluated edoxaban, 6 examined rivaroxaban, and 6 assessed apixaban. All 11 articles were included in the present meta-analysis. Figure 1 illustrates the literature review and article selection process.

### Baseline characteristics

The 11 studies had a combined total of 2226 patients. In the DOAC group, only direct Xa inhibitors were used, with 165 patients given edoxaban [20, 27], 368 receiving rivaroxaban [11, 21–23, 27–29], and 412 using apixaban [23–29]. However, 140 patients had no details of their DOAC subtype [27, 29]. As for LMWHs, 1141 patients received them. Dalteparin was used with 693 patients, enoxaparin with 447 patients, and nadroparin with 1 patient [11, 20–29].

Regarding the type of GI cancer, 526 patients had upper GI cancer (cancer of the esophagus or stomach), 945 had lower GI cancer (cancer of the colon or rectum), 740 had hepatobiliary-pancreatic cancer (hepatocellular carcinoma, cholangiocarcinoma, cancer of the gallbladder, or pancreatic cancer), and 7 had neuroendocrine tumors. These patients were also subdivided into 3 groups. Group 1 had 1471 patients with luminal GI cancer (cancer of the esophagus, stomach, colon, or rectum) [11, 20–29]. Group 2 had 740 patients with nonluminal GI cancer (hepatocellular carcinoma, cancer of the gallbladder, or pancreatic cancer) [11, 20–29]. Group 3 had 7 patients with neuroendocrine tumors [23].

**Fig. 2:**
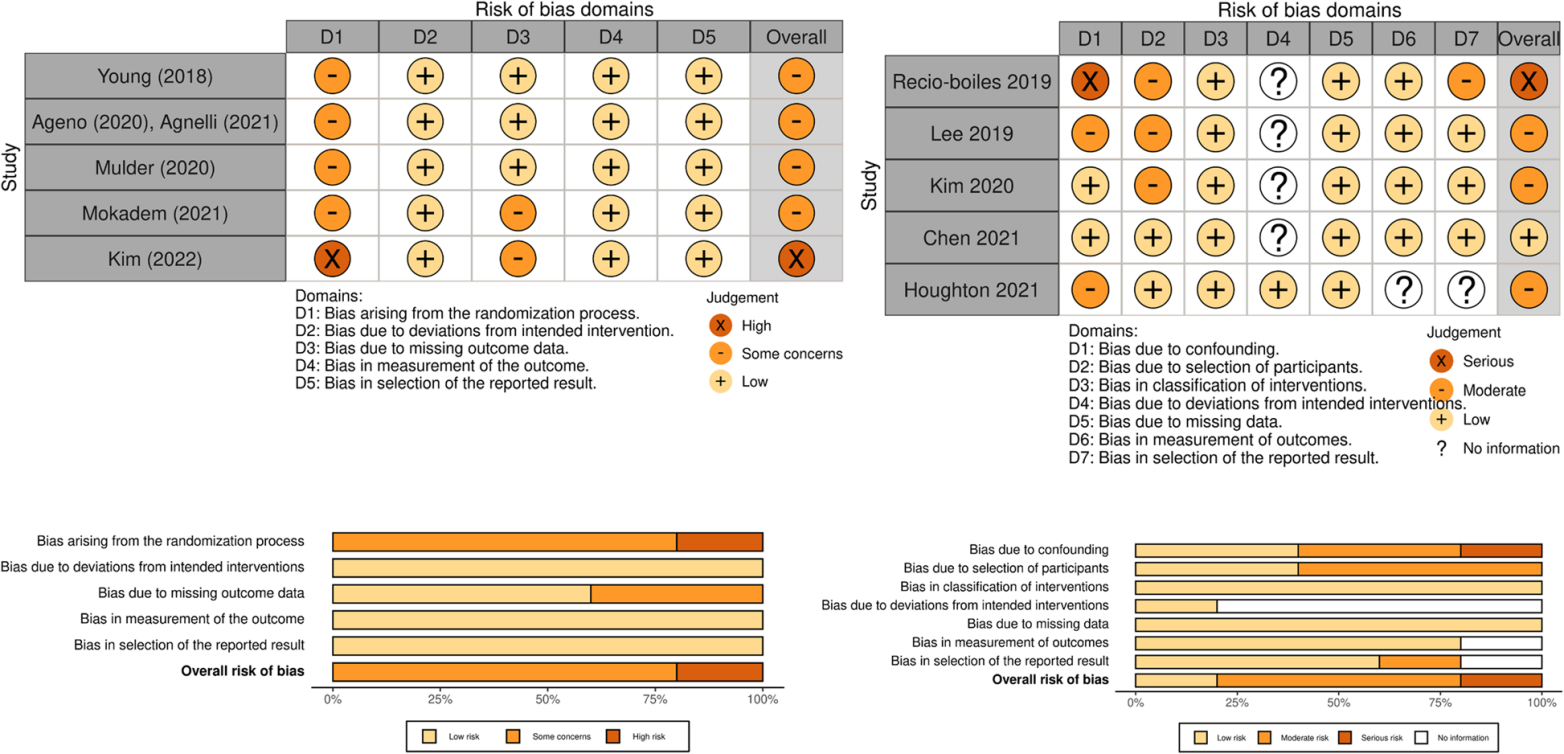
Risk of bias plot in included randomized and nonrandomized studies

The studies’ follow-up periods ranged from 6 to 12 months [11, 20–29]. The characteristics of the recruited patients are summarized in Table 1, while Fig. 2 presents the risk-of-bias plot of the studies.

**Table 1:** Characteristics of the patients in the 11 studies included in this meta-analysis

**Fist author and year of publication**	**Patients inclusion criteria**	**Group of treatment (No.)**	**Dose of anticoagulants**	**Type of GI cancers (No.)**	**Type of GI cancers (luminal VS non-luminal, others) (No.)**	**Follow up time in months (study period)**	**Type of study**
Young et al 2018 [11]	Patients with active cancer (diagnosis or treatment within 6 months, recurrent or metastatic cancer receiving rivaroxaban or LMWH for symptomatic PE, DVT or incidental PE	Rivaroxaban (91)	15 mg twice daily for 3 weeks then 20 mg daily	Esophagus (11) Stomach (4) Colorectal (55) HB (2) Pancreas (19)	Luminal (70) Non-luminal (21)	6 months September 2013-December 2016)	Randomized controlled trial
		Dalteparin (86)	200 IU/Kg once daily for 30 days then 150 IU/Kg	Esophagus (19) Stomach (7) Colorectal (47) HB (2) Pancreas (11)	Luminal (73) Non-luminal (13)		
Recio-Boiles et al. 2019 [23]	Patients receiving DOACs or LMWHs with GI cancer and symptomatic or incidental VTE	Rivaroxaban (37)	15 mg twice daily for 3 weeks then 20 mg daily	Esophagus (3) Stomach (4) Colorectal (26) HB (1) Pancreas (28) NET (4)	Luminal (33) Non-luminal (29) NET (4)	6 months up (November 2013-February 2017)	Retrospective cohort study
		Apixaban (29)	10 mg twice daily for 7 days then 5 mg twice daily				
		Enoxaparin (40)	1 mg/kg/dose twice daily or 1.5 mg/kg once daily	Esophagus (0) Stomach (5) Colorectal (11) HB (6) Pancreas (15) NET (3)	Luminal (16) Non-luminal (21) NET (3)		
Lee et al. 2019 [21]	Patients receiving rivaroxaban or LMWHs with GI cancer and confirmed PE or DVT	Rivaroxaban (78)	15 mg twice daily for 3 weeks then 20 mg daily	Stomach (19) Colorectal (21) Pancreato-biliary (38)	Luminal (40) Non-luminal (38)	6 months (January 2012-December 2016)	Retrospective cohort study
		LMWH (203) -Dalteparin (177) -Enoxaparin (25) -Nadroparin (1)	Dalteparin: 200 IU/kg once daily Enoxaparin: 1 mg/kg/dose twice daily Nadroparin: 85.5 IU/kg twice daily	Stomach (98) Colorectal (11) Pancreato-biliary (94)	Luminal (109) Non-luminal (94)		
Ageno et al. 2020 [24] and Agnelli et al. 2021 [25] Caravaggio study	Patients with active cancer or diagnosed within 2 years receiving apixaban or dalteparin for symptomatic or incidental PE/DVT	Apixaban (188)	10 mg twice daily for 7 days then 5 mg twice daily	Esophagus (13) Stomach (8) HB (11) Pancreas (33) Colorectal (120) Unknown (3)	Luminal (141) Non-luminal (44)	6 months (April 2017-June 2019)	Randomized controlled trial (non-inferiority trial)
		Dalteparin (187)	200 IU/Kg once daily for 30 days then 150 IU/Kg	Esophagus (10) Stomach (17) HB (9) Pancreas (34) Colorectal (112) Unknown (5)	Luminal (139) Non-luminal (43)		
Mulder et al. 2020 [20]	Cancer patients with symptomatic or incidental PE/DVT receiving edoxaban or LMWH	Edoxaban (165)	60 mg once daily after initial LMWH 5 days (30 mg once daily in creatinine clearance 30–50 mL/min, BM below 60 kg or concomitant treatment with potent P-glycoprotein inhibitors)	Esophagus (23) Stomach (10) Colorectal (83) HB (14) Pancreas (35)	Luminal (116) Non-luminal (49)	6 months (July 2015-December 2016)	Randomized controlled trial (non-inferiority trial)
		Dalteparin (140)	200 IU/Kg once daily for 30 days then 150 IU/Kg	Esophagus (11) Stomach (10) Colorectal (79) HB (12) Pancreas (28)	Luminal (100) Non-luminal (40)		
Kim et al. 2020 [22]	Patients with upper GI tract and HBP cancer receiving LMWH or rivaroxaban (including unresectable or metastatic cancer)	Rivaroxaban (69)	15 mg twice daily for 3 weeks then 20 mg daily	Esophagus (1) Stomach (23) HB (18) Pancreas (27)	Luminal (24) Non-luminal (45)	6 months (January 2004-December 2014)	Retrospective cohort study
		LMWH (105) -Dalteparin (57) -Enoxaparin (48)	Dalteparin: 200 IU/Kg once daily for 30 days then 150 IU/Kg Enoxaparin: 1 mg/kg twice daily	Esophagus (7) Stomach (52) HB (21) Pancreas (25)	Luminal (59) Non-luminal (46)		
Mokadem et al. 2021 [26]	Patients with active malignancy presenting with acute deep venous thrombosis and still treated with chemotherapy	Apixaban (25)	10 mg twice daily for 7 days then 5 mg twice daily	Colorectal (23) Liver (2)	Luminal (23) Non-luminal (2)	6 months (July 2019-June 2020)	Randomized controlled trial
		Enoxaparin (23)	1 mg/kg twice daily	Colorectal (19) Liver (4)	Luminal (19) Non-luminal (4)		
Chen et al. 2021 [27]	Patients aged 18 years or older with active cancer who developed newly diagnosed VTE	DOACs (96)	Apixaban: 5 mg twice daily Edoxaban: 60 mg once daily Dabigratan: 150 mg twice daily Rvaroxaban: 15 mg twice for the first 21 days and then 20 mg once daily	Esophagus (8) Stomach (8) Colorectal (80)	Luminal (96)	12 months (January 2012-January 2019)	Population-based cohort study
		Enoxaparin (122)	1 mg/kg twice daily	Esophagus (9) Stomach (38) Colorectal (75)	Luminal (122)		
Houghton et al. 2021 [28]	Consecutive GI cancer patients with acute cancer associated VTE	Apixaban (170) Rivaroxaban (93)	Not available	Upper GI (29) Lower GI (103) Pancreas (103) Hepatobiliary (28)	Luminal (132) Non-luminal (131)	3 and 6 months (March 2013-April 2020)	Prospective cohort study
		Enoxaparin (189)		Upper GI (29) Lower GI (79) Pancreas (59) Hepatobiliary (22)	Luminal (108) Non-luminal (81)		
Kim et al. 2022 [29]	Patients aged 19–80 years old, with histologically confirmed, advanced upper GI tract, hepatobiliary, and pancreatic cancer, and newly diagnosed (within 2 weeks before randomization) symptomatic or incidental VTE	Apixaban or Rivaroxaban (44)	Apixaban: 10 mg twice daily for 7 days then 5 mg twice daily Rivaroxaban: 15 mg twice daily for 3 weeks then 20 mg daily	Esophagus (8) Stomach (19) Colorectal (0) Pancreas (6) Hepatobiliary (11)	Luminal (27) Non-luminal (17)	Every 1–4 weeks, and then followed up every 3 months for 1 year or until death (August 2017-June 2020)	Randomized controlled trial
		Dalteparin (46)	200 IU/Kg once daily for 30 days then 150 IU/Kg	Esophagus (5) Stomach (18) Colorectal (1) Pancreas (12) Hepatobiliary (10)	Luminal (24) Non-luminal (22)		

Abbreviations: *VTE* Venous thromboembolism, *PE* Pulmonary embolism, *DVT* Deep vein thrombosis, *GI* Gastrointestinal, *HB* Hepatobiliary, *NET* Neuroendocrine tumor, *LMWH* Low molecular weight heparin

### Clinical bleeding outcome

**Fig. 3:**
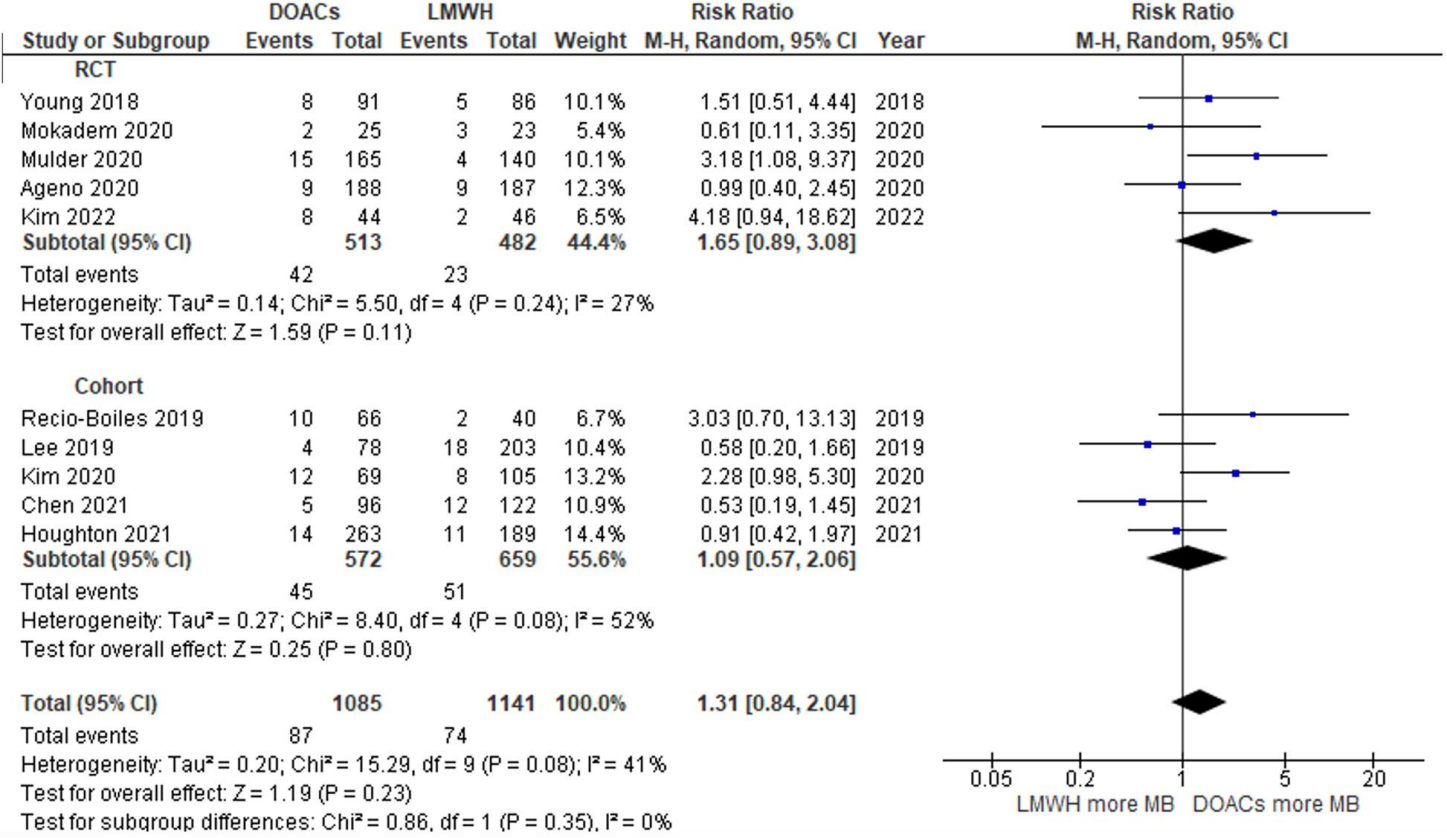
Forest plot of studies that compared major bleeding of the DOAC and LMWH groups

Six randomized controlled trials and 5 retrospective studies compared DOACs with LMWHs. Major bleeding was defined according to the ISTH criteria [15]; in the Caravaggio study, it was combined with “bleeding resulting in surgical intervention” [13]. Our pooled analysis showed a nonsignificantly higher risk of major bleeding in patients receiving DOACs than in those receiving LMWHs, with a pooled relative risk (RR) of 1.31. However, the pooled effect estimate did not reach statistical significance (95% CI: 0.84–2.04; *P* = 0.23). Furthermore, the heterogeneity of the meta-analysis was low, with an I2 value of 41% (Fig. 3) [11, 20–24, 26–29].

**Fig. 4:**
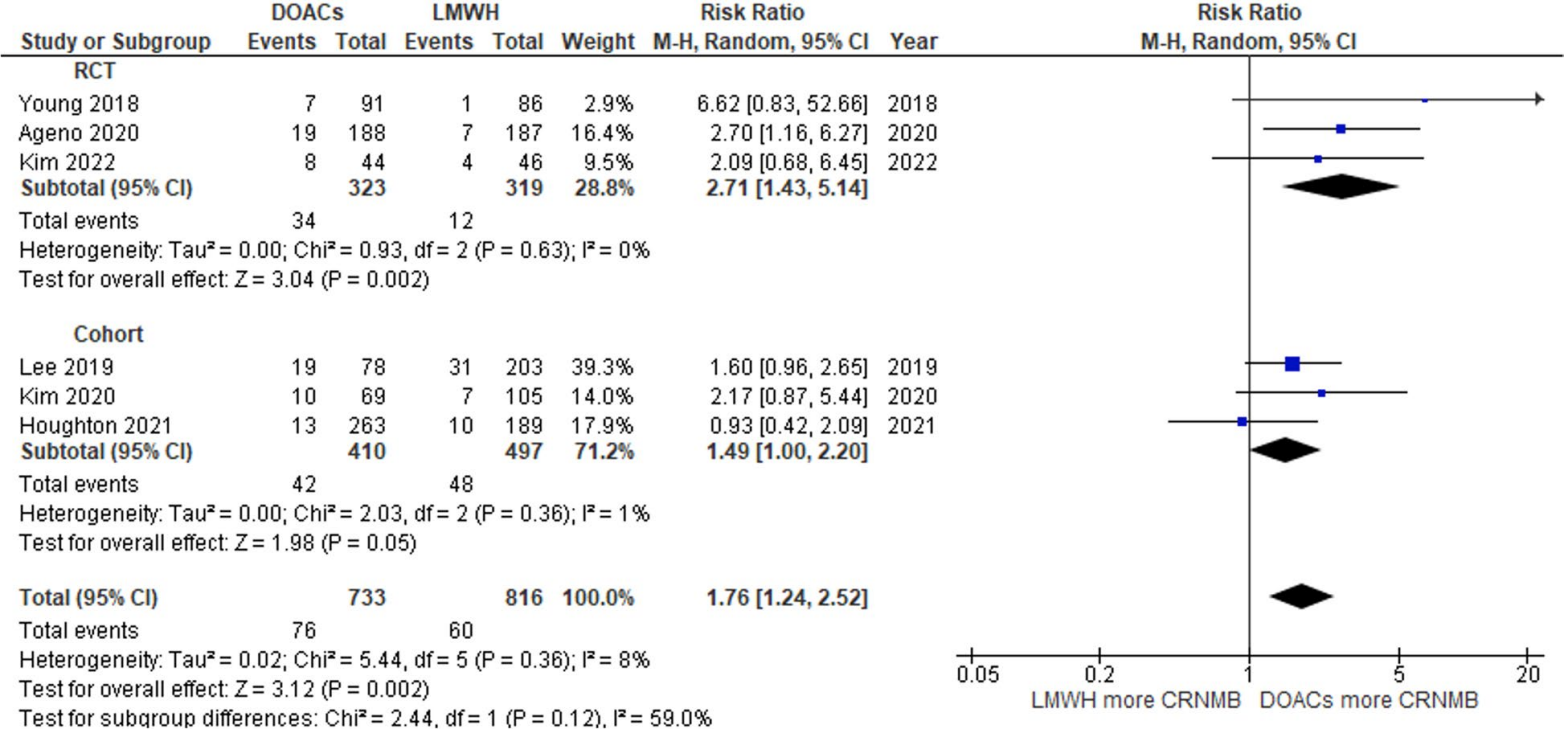
Forest plot of studies that compared clinically relevant nonmajor bleeding (CRNMB) of the DOAC and LMWH groups

In contrast, the incidence of CRNMB was significantly higher in the DOAC group than in the LMWH group, with a pooled RR of 1.76 (95% CI: 1.24–2.52; *P* = 0.002; I2 = 8%; Fig. 4) [11, 21, 22, 24, 28, 29].

### Location of bleeding

Four studies reported the locations of major bleeding in patients with GI cancer treated with DOACs [22, 24, 29, 30]. Of 50 bleeding events, 41 occurred in the GI tract. The central nervous system, genitourinary tract, retro- and intraperitoneal areas, upper airway, epistaxis, vagina, and muscle hematoma were other bleeding sites. The details of major bleeding and the type of anticoagulant therapy are listed in Table 2.

**Table 2:** Major bleeding details and type of anticoagulant therapy reported by studies included in this meta-analysis

References	Group of treatment (No. of bleeding patients)	Number of events and the site of major bleeding						
		**Upper GI tract**	**Lower GI tract**	**Central nervous system**	**Genitourinary tract**	**Retroperitoneal area**	**Intra-abdominal area**	**Other sites**
Kraaipoel et al. 2018 [30]	Edoxaban (21)	16	3	-	-	1	-	1 Epistaxis
	Dalteparin (5)	1	-	2 intracerebral hemorrhage 1 thoracic spinal cord	-	-	-	1 Not mentioned
Kim et al. 2020 [22]	Rivaroxaban (12)	7	2	-	-	-	-	3 Unspecified GI tract
	LMWHs (8)	2	1	-	-	-	3 hemoperitoneum	1 Unspecified GI tract 1 Unspecified site
Ageno et al. 2020 [24]	Apixaban (9)	4	3	-	1	-	1	
	Dalteparin (9)	3	3	-	-	1	-	2 Upper airway 1 Muscle
Kim et al. 2022 [29]	Apixaban or Rivaroxaban (6)	6		1	-	-	-	1 Vaginal
	Dalteparin (2)	2		-	-	-	-	

Abbreviations: *GI* Gastrointestinal, *LMWHs* Low molecular weight heparins

### Recurrent VTE outcome

**Fig. 5:**
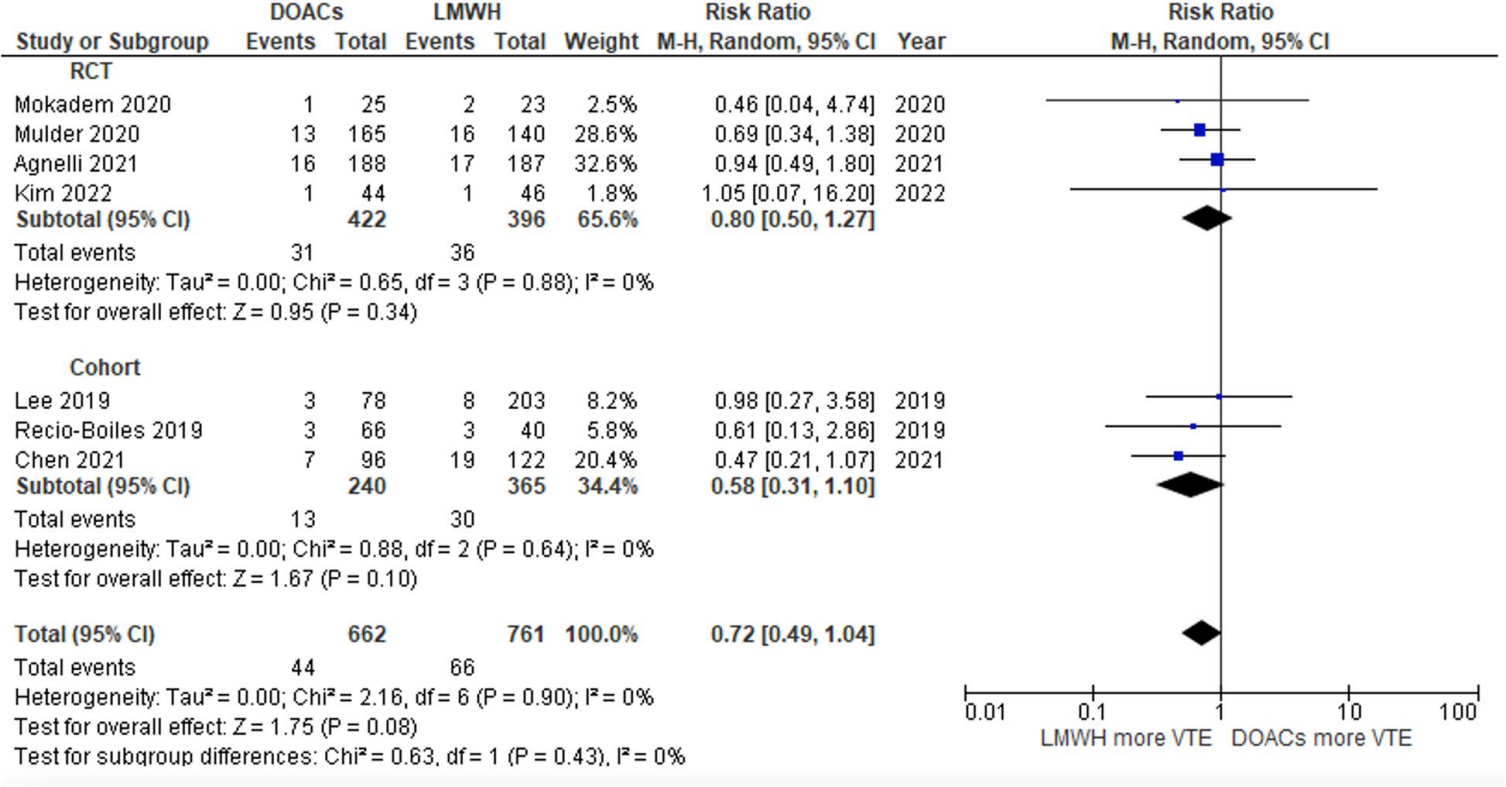
Forest plot of studies that compared recurrent VTE of the DOAC and LMWH groups

The rates of recurrent VTE in patients who received DOACs and those who received LWMHs were not significantly different, with a pooled RR of 0.72 (95% CI: 0.49–1.04; *P* = 0.08; I^2^ = 0%; Fig. 5) [20, 21, 23, 25–27, 29].

### Subgroup analysis of outcomes by type of GI cancer

**Fig. 6:**
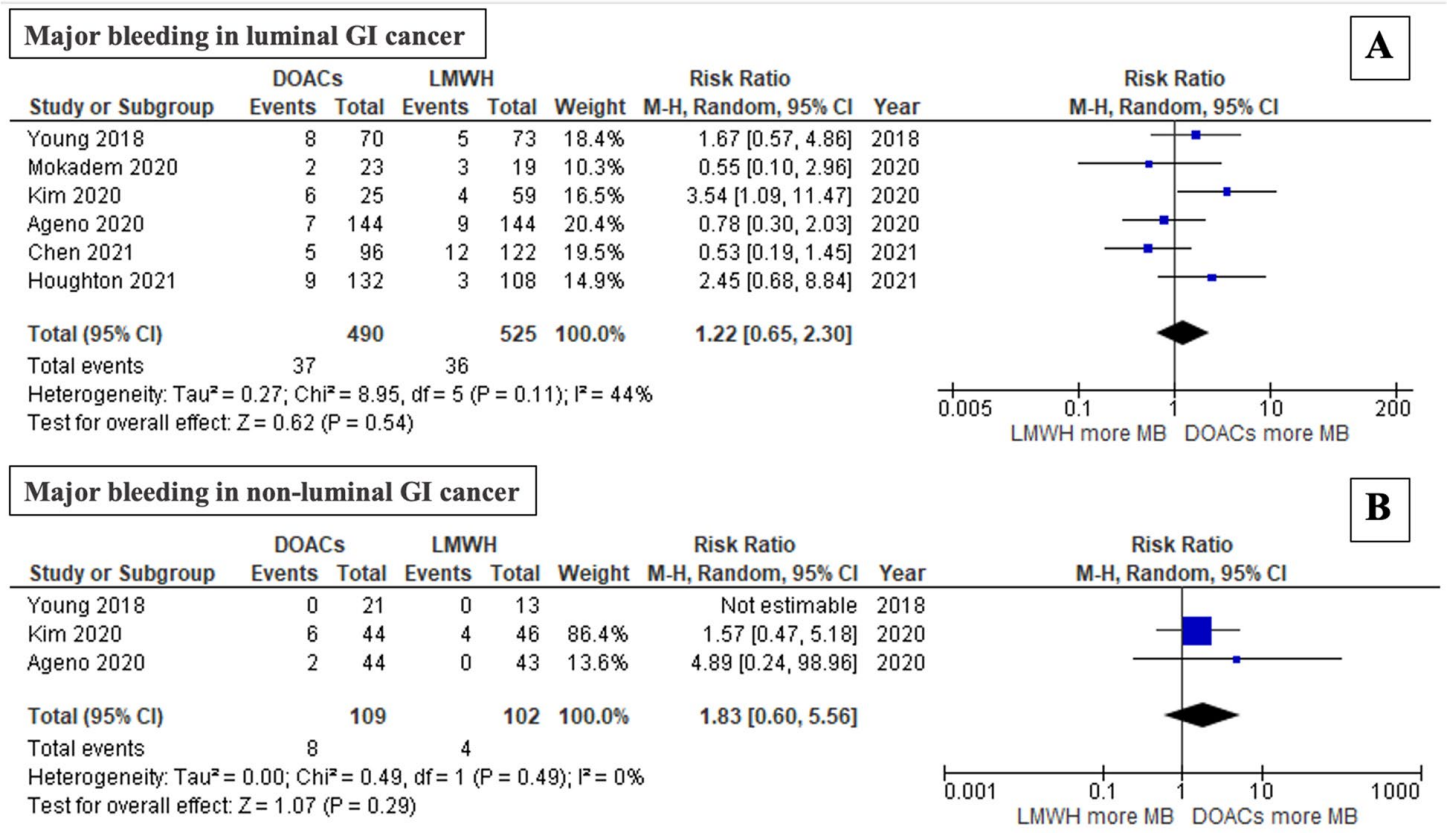
Forest plot of studies that compared (**A**) major bleeding in luminal GI cancer patients and (**B**) major bleeding in nonluminal GI cancer patients

A subgroup analysis evaluating major bleeding events in patients with luminal and nonluminal GI cancer revealed a trend toward nonsignificantly increased major bleeding in patients with luminal GI cancer treated with DOACs, with a pooled RR of 1.22 (95% CI: 0.65–2.30; *P* = 0.54; I^2^ = 44%; Fig. 6A) [11, 22, 24, 26–28]. Similarly, among nonluminal GI cancer patients, major bleeding was not significantly different between groups. However, the patients who received DOACs showed a trend toward more major bleeding, with a pooled RR of 1.83 (95% CI: 0.60–5.56; *P* = 0.29; I2 = 0%; Fig. 6B) [11, 22, 24].

### Subgroup analysis of outcomes by type of study

Both RCTs and cohort studies were included in this current systematic review and meta-analysis to analyze bleeding outcomes based on the type of study [11, 20–24]. In the case of the RCT studies, the trend of major bleeding outcomes was similar to the pooled analysis. The pooled RRs of major bleeding were 1.65 (95% CI: 0.89–3.08; *P* = 0.11; I2 = 27%; Fig. 3) [11, 20, 24, 26, 29]. The rate of CRNMB was significantly higher in the DOAC group, with a pooled RR of 2.71 (95% CI: 1.43–5.14; *P* = 0.002; I2 = 0%; Fig. 4) [11, 24]. The pooled RRs of major bleeding and CRNMB in cohort studies were comparable to the full-analysis results (Figs. 3 and 4) [21–23, 27, 28]. Likewise, the pooled RR of VTE recurrence from the RCTs and cohort studies was not different between the DOAC and LMWH groups (Fig. 5) [20, 21, 23, 25–27, 29].

### Subgroup analysis of bleeding risk by DOAC type

**Fig. 7:**
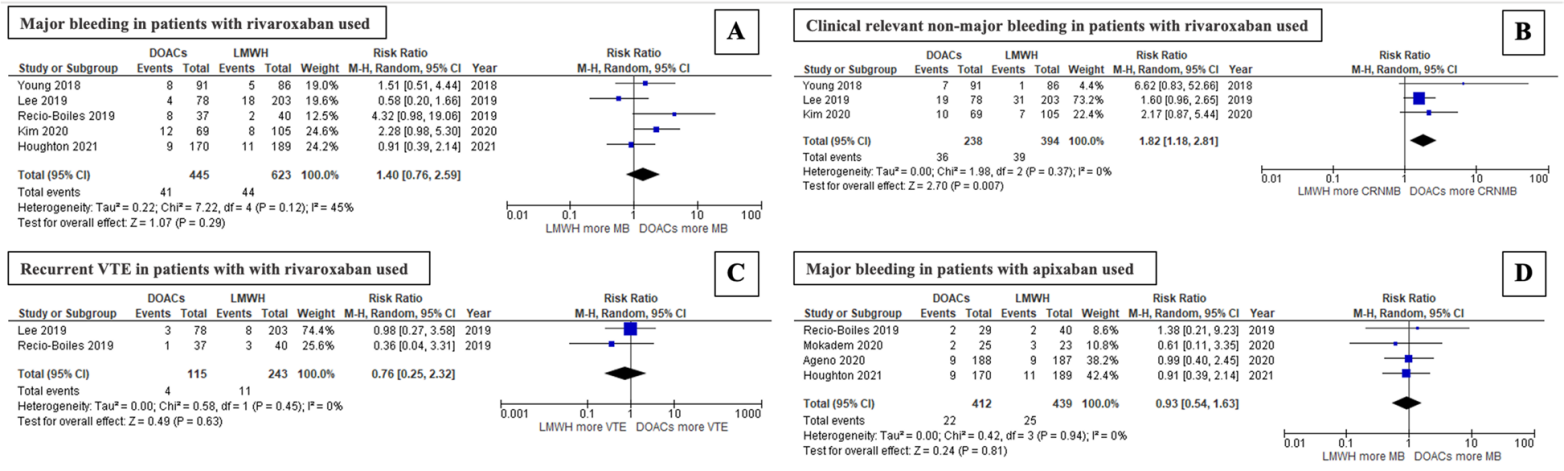
Forest plot of studies that compared (**A**) major bleeding in patients treated with rivaroxaban, (**B**) clinically relevant nonmajor bleeding (CRNMB) in patients treated with rivaroxaban, (**C**) recurrent VTE in patients treated with rivaroxaban, and (**D**) major bleeding in patients treated with apixaban in the DOAC and LMWH groups

Neither the rivaroxaban nor the apixaban subgroup was associated with a significant increase in major bleeding events compared with the LMWH arm. For the rivaroxaban group, the pooled RR was 1.40 (95% CI: 0.76–2.59; *P* = 0.29; I2 = 45%; Fig. 7A) [11, 21–23, 28], while for the apixaban group, the pooled RR was 0.93 (95% CI: 0.54–1.63; *P* = 0.81; I2 = 0%; Fig. 7D) [23, 24, 26, 28]. In contrast, CRNMB rates were significantly higher for patients treated with rivaroxaban than for those treated with LMWHs (pooled RR: 1.82; 95% CI: 1.18–2.81; *P* = 0.007; I2 = 0%; Fig. 7B) [11, 21, 22]. However, there was no significant difference between the rates of recurrent VTE of the 2 groups (pooled RR: 0.76; 95% CI: 0.25–2.32; *P* = 0.63; I2 = 0%; Fig. 7C) [21, 23]. Figure 7 presents a forest plot of studies that compared major bleeding, CRNMB, and recurrent VTE in patients who received each DOAC compared with LMWHs.

Due to the limited number of comparative studies of apixaban and LMWHs in GI cancer patients, data specific to CRNMB and recurrent VTE could not be demonstrated. Likewise, analysis of major bleeding, CRNMB, and recurrent VTE could not be performed for the subgroup of GI cancer patients receiving edoxaban due to insufficient data comparing edoxaban and LMWHs.

### Quality assessment

With the randomized controlled studies, the risk-of-bias assessment revealed some concerns for 4 studies and a high risk of bias for 1 study concerning allocation concealment. Most of the risk-of-bias assessments of the observational studies were moderate, with only 1 study having a serious risk. The risks were related to confounding factors, participant selection, and lack of deviation from the intended intervention report.

## Discussion

Several studies have demonstrated the efficacy and safety of DOACs in patients with cancer-associated venous thromboembolism [10–13]. As a result, DOACs have become an alternative to LMWHs for the treatment of CAT. Despite the noninferior efficacy of DOACs to LMWHs for preventing recurrent VTE, higher bleeding risks were found with certain DOACs than with LMWHs in subgroup analyses of patients with GI and genitourinary tract cancers [30–32]. However, previous randomized controlled trials enrolled patients with various kinds of cancer. Thus, there is a need for a systematic review and meta-analysis that focuses on DOACs for treating acute venous thromboembolism in patients with gastrointestinal cancer.

The pooled analysis found no significant differences in the major bleeding or the recurrent VTE of the patients receiving DOACs and patients given LMWHs. In addition, major bleeding was similar in the subgroup analysis that compared luminal and nonluminal GI malignancies. In contrast, the rate of CRNMB was significantly higher for patients in the DOAC group than in the LMWH group.

A previous randomized controlled trial of VTE treatment in noncancer patients demonstrated a higher incidence of GI bleeding among patients treated with rivaroxaban than among those treated with warfarin [31]. Moreover, in the SELECT-D study, GI hemorrhage and CRNMB were significantly higher in the rivaroxaban group than in the LMWH group [11]. The Hokusai VTE Cancer trial found a higher rate of major bleeding—but not CRNMB—in patients with cancer receiving edoxaban than in those receiving dalteparin. A higher rate of GI bleeding was also observed in patients with GI cancer [10]. In contrast, 2 studies reported no significant difference in the risk of major GI bleeding in patients with cancer receiving apixaban and those receiving LMWHs [12, 13].

Interestingly, the analysis of bleeding risk and the DOAC type used for acute VTE showed no significant differences in major bleeding in the rivaroxaban and apixaban subgroups. This result suggests that the DOAC type might not be the only high-risk factor for bleeding in patients with GI cancer. Nonetheless, this meta-analysis observed higher CRNMB in rivaroxaban patients than in LMWH patients.

The meta-analysis results are consistent with previous meta-analyses of DOAC use in cancer patients. Those studies reported higher CRNMB [32] but similar major bleeding events [33, 34] in DOAC users compared with those taking LMWHs. Although the current meta-analysis found no significant difference in the major bleeding rates of patients receiving DOACs and those administered LMWHs, there was a trend toward increased major bleeding in the DOAC group. Moreover, the efficacy of DOACs for preventing recurrent VTE did not differ from that of LMWH. Therefore, DOACs should be considered an effective alternative treatment to LMWH for treating acute VTE, with no statistically significant difference in major bleeding among patients with GI malignancies. However, the significantly higher CRNMB associated with DOACs must be considered when deciding to use DOACs for GI cancer patients. The risk of bleeding should be disclosed and discussed with patients before starting therapy.

Recently, Hussain et al.performed a meta-analysis of the risk of overall bleeding and recurrent VTE in cancer-associated thrombosis treated with factor Xa inhibitors compared with patients treated with LMWHs [35]. However, their meta-analysis had only 3 observational studies in the subgroup analysis of patients with GI cancer [35]. In contrast, our meta-analysis examined 11 studies on patients with GI cancer. Subgroup analyses based on the GI-cancer and DOAC types were also conducted. Analysis for consistency among studies based on visual inspection of forest plots and the low I^2^ values showed no or low heterogeneity.

This study has some limitations. First, the low number of events and included patients may preclude statistically significant differences in some outcomes, such as recurrent VTE. Second, data were lacking on some baseline patient characteristics that might affect the risk of thrombosis (such as sex, age, cancer treatment, and patient status [inpatient or outpatient]). Third, the definitions of the primary outcomes varied among the included studies. Fourth, only 3 studies included recurrent thrombosis as a primary outcome. Fifth, due to the limited number of studies in the meta-analysis, analytical investigation of heterogeneity could not be evaluated. Last, publication bias could also not be assessed due to the limited number of studies.

## Conclusions

The pooled data from this meta-analysis suggest that the efficacy of DOACs for the prevention of recurrent VTE in patients with GI malignancies is comparable to that of LMWHs. Treatment of acute VTE with DOACs is associated with a significantly increased risk of CRNMB but not with a major bleeding risk. Therefore, the benefits and risks of DOAC treatment should be discussed with patients with GI cancer before commencing therapy.

## Supplementary Information

**Additional file 1.** Search Strategy

**Additional file 2.** PRISMA

**Additional file 3.** Definitions of clinically relevant nonmajor bleeding used by the 11 studies included in this meta-analysis

## Data Availability

The datasets used or analyzed during the current study are available from the corresponding author on reasonable request.

## Abbreviations

CAT: Cancer-associated thrombosis
CI: Confidence interval
CRNMB: Clinically relevant nonmajor bleeding
DOACs: Direct oral anticoagulants
GI: Gastrointestinal
ISTH: International Society on Thrombosis and Haemostasis
LMWH: Low-molecular-weight heparin
MB: Major bleeding
RCT: Randomized controlled trial
RR: Relative risk
VTE: Venous thromboembolism
