# Supplementary material for: Direct oral anticoagulants versus low-molecular-weight heparins for the treatment of acute venous thromboembolism in patients with gastrointestinal cancer: a systematic review and meta-analysis
Source: Thromb J. 2022 Jul 28;20:41. doi: 10.1186/s12959-022-00399-7 (PMC9330678; doi:10.1186/s12959-022-00399-7)
Supplement: Supplementary file 1 — Additional file 1. Search Strategy [file 12959_2022_399_MOESM1_ESM.docx]

**Supplementary Data 1**–Search strategy

**EMBASE Database**

1. ‘direct oral anticoagulant' /exp OR ‘direct oral anticoagulant'
2. ‘DOACS’/exp OR ‘DOACS’
3. ‘NOACS’/exp OR ‘NOACS’
4. ‘novel anticoagulant'’/exp OR ‘novel anticoagulant'’
5. ‘apixaban /exp OR ‘apixaban
6. ‘rivaroxaban’/exp OR ‘rivaroxaban’
7. ‘dabigatran /exp OR ‘dabigatran
8. ‘edoxaban’/exp OR ‘edoxaban’
9. ‘gastrointestinal’/exp OR ‘gastrointestinal’
10. / gastric’/exp OR ‘gastric’
11. ‘stomach’/exp OR ‘stomach’
12. ‘colon’/exp RO ‘colon’
13. ‘intestinal’/exp OR ‘intestinal'’
14. ‘rectal’/exp OR ‘rectal’
15. ‘hepatobiliary’/exp OR ‘hepatobiliary’
16. ‘pancreatic’/exp OR ‘pancreatic’
17. ‘cancer’/exp OR ‘cancer’
18. ‘malignancy’/exp OR ‘malignancy’
19. #1 OR #2 OR #3 OR #4 OR #5 OR #6 OR #7 OR #8
20. #9 OR #10 OR #11 OR #12 OR #13 OR #14 OR #15 OR #16
21. #17 OR #18
22. #19 AND #20 AND #21

**Ovid MEDLINE Database**

1. direct oral anticoagulant.mp
2. DOACS.mp.
3. NOACS.mp.
4. novel anticoagulant.mp.
5. apixaban.mp.
6. rivaroxaban.mp.
7. dabigratan.mp.
8. edoxaban.mp.
9. gastrointestinal.mp.
10. gastric.mp.
11. stomach.mp.
12. colon.mp.
13. intestinal.mp.
14. rectal.mp.
15. hepatobiliary.mp.
16. pancreatic.mp.
17. cancer.mp.
18. malignancy.mp.
19. 1 or 2 or 3 or 4 or 5 or 6 or 7 or 8
20. 9 or 10 or 11 or 12 or 13 or 14 or 15 or 16
21. 17 or 18
22. 19 and 20 and 21
