# Supplementary figures and images for: Direct oral anticoagulants versus low-molecular-weight heparins for the treatment of acute venous thromboembolism in patients with gastrointestinal cancer: a systematic review and meta-analysis
Source: Thromb J. 2022 Jul 28;20:41. doi: 10.1186/s12959-022-00399-7 (PMC9330678; doi:10.1186/s12959-022-00399-7)

**Supplementary data 2: PRISMA**


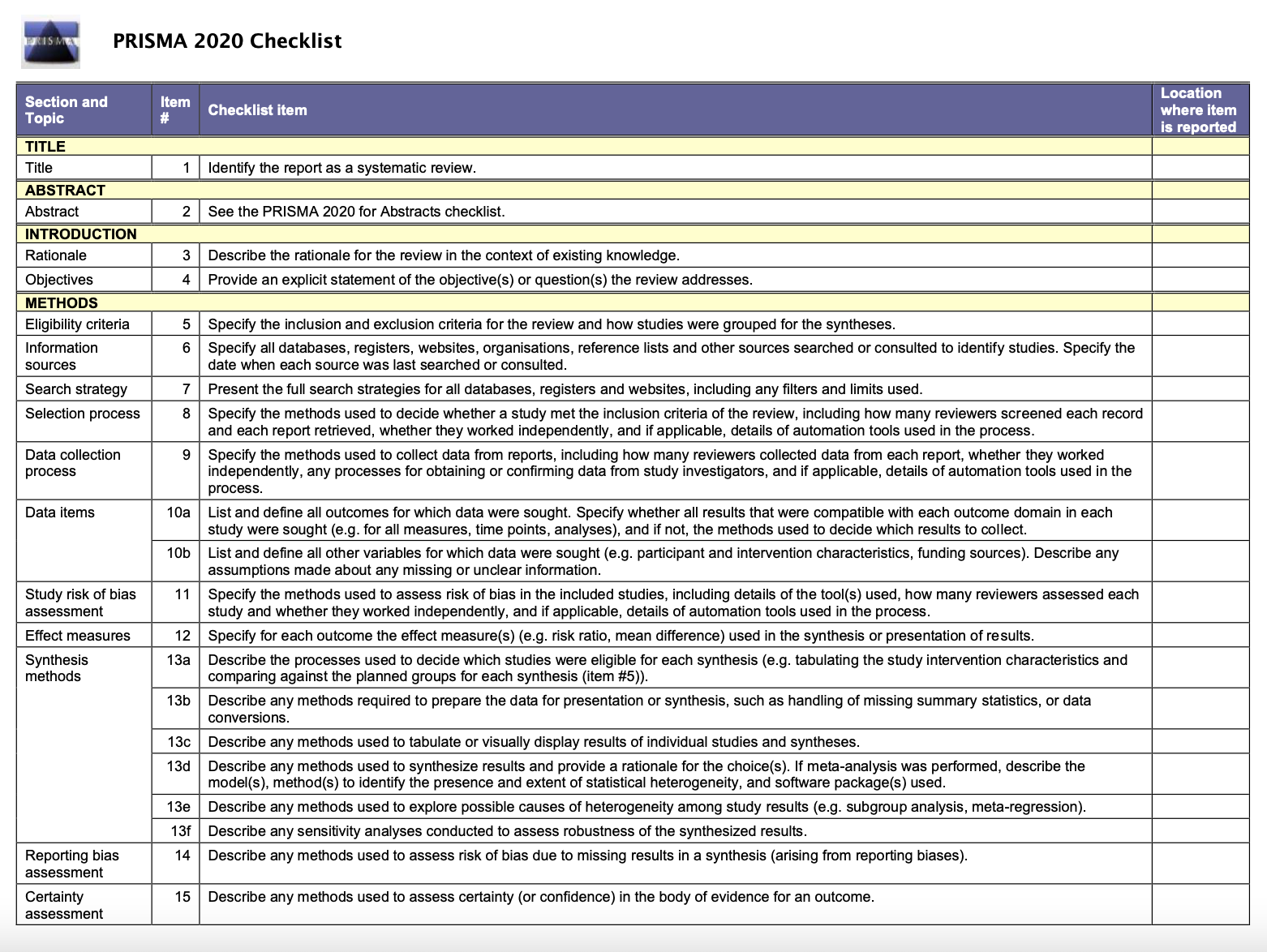


**4**

**4-5**

**4-5**

**4-5**

**4-5**

**4-5**

**4-5**

**4-5**

**5**

**5**

**5**

**4-5**

**4-5**

**4**

**4**

**4**

**4**

**4**

**2**

**3**

**1**


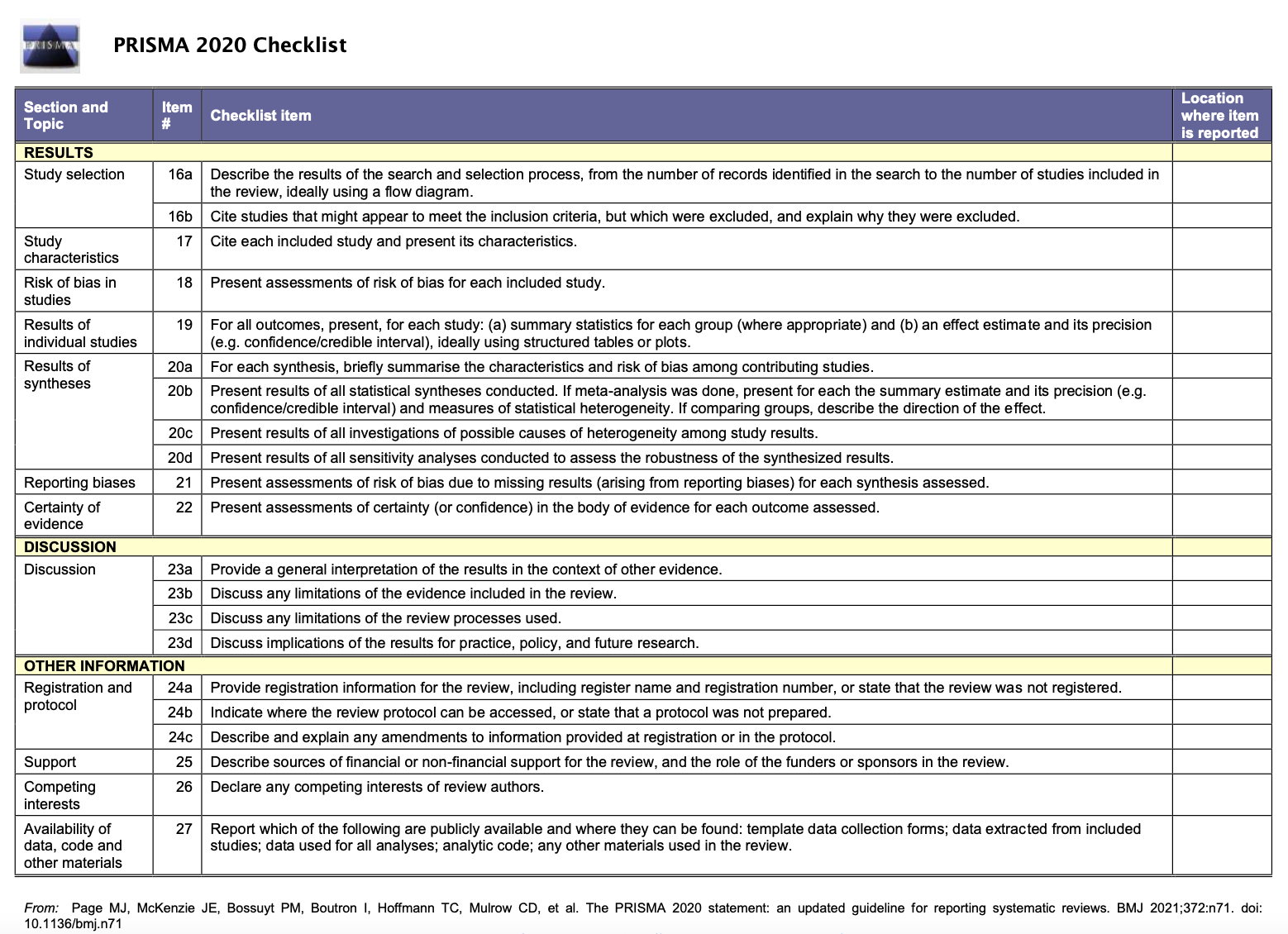


**13**

**13**

**13**

**13**

**13**

**4**

**9-11**

**9-11**

**9-11**

**9-11**

**7-9**

**9**

**-**

**7-9**

**7-9**

**7-9**

**Fig.2**

**6**

**6**

**6**

**9**

Supplement: Supplementary file 2 — Additional file 2. PRISMA [file 12959_2022_399_MOESM2_ESM.docx]
