# Supplementary material for: Direct oral anticoagulants versus low-molecular-weight heparins for the treatment of acute venous thromboembolism in patients with gastrointestinal cancer: a systematic review and meta-analysis
Source: Thromb J. 2022 Jul 28;20:41. doi: 10.1186/s12959-022-00399-7 (PMC9330678; doi:10.1186/s12959-022-00399-7)
Supplement: Supplementary file 3 — Additional file 3. Definitions of clinically relevant nonmajor bleeding used by the 11 studies included in this meta-analysis [file 12959_2022_399_MOESM3_ESM.docx]

**Supplementary Data 3**

**Definitions of clinically relevant nonmajor bleeding used by the 11 studies included in this meta-analysis**

| Studies | Definition |
| --- | --- |
| Young et al. [11] | Acute, clinically overt episodes that did not meet major criteria but required medical intervention, unscheduled contact with a physician, interruption or discontinuation of the study drug, discomfort, or impairment of activities of life |
| Kim et al. [23] |  |
| Ageno et al. [25] | CRNMB events were defined as acute clinically overt bleeding that did not meet the criteria for major bleeding, but required nonsurgical, medical intervention by a health care professional, leading to hospitalization or increased level of care, or prompting evaluation. |
| Lee et al. [22] | Any obvious bleeding occurring during treatment with LMWH or rivaroxaban and resulted in medical intervention, unscheduled visits with clinicians, discontinuance of anticoagulants, or a decline in the activities of daily life. |
| Houghton et al. [28] | Overt bleeding not meeting the criteria for major bleeding but associated with medical intervention, unscheduled contact with a member of the health care team, or temporary cessation of treatment |
| Kim et al. [29] | Acute and clinically overt bleeding with one or more of the following: the need for medical intervention, unscheduled contact with a physician, interruption or discontinuation of anticoagulation, or impairment of activities of daily life |
